# Identification of two novel *Rhizoctonia solani*-inducible *cis*-acting elements in the promoter of the maize gene, *GRMZM2G315431*

**DOI:** 10.1038/srep42059

**Published:** 2017-02-06

**Authors:** Ning Li, Jing Chen, Fangfang Yang, Shutong Wei, Lingguang Kong, Xinhua Ding, Zhaohui Chu

**Affiliations:** 1 State Key Laboratory of Crop Biology, College of Agronomy, Shandong Agricultural University, Tai’an, 271018, Shandong, PR China; 2 Shandong Provincial Key Laboratory of Agricultural Microbiology, College of Plant Protection, Shandong Agricultural University, Tai’an, 271018, Shandong, PR China

## Abstract

Plants are continuously exposed to myriad pathogen stresses. However, the molecular mechanisms by which these stress signals are perceived and transduced are poorly understood. In this study, the maize gene *GRMZM2G315431* was identified to be highly inducible by *Rhizoctonia solani* infection, suggesting that the promoter of *GRMZM2G315431* (pGRMZM2G315431) might contain a specific *cis*-acting element responsive to *R. solani* attack. To identify the *R. solani*-responsive element in pGRMZM2G315431, a series of binary plant transformation vectors were constructed by fusing pGRMZM2G315431 or its deletion-derivatives with the reporter genes. In the transient gene expression system of *Nicotiana benthamiana* leaves inoculated with *R. solani*, GUS quantification suggested that the DNA fragment contains the unknown pathogen-inducible *cis-*elements in the −1323 to −1212 region. Furthermore, detailed quantitative assays showed that two novel *cis*-elements, GTTGA in the −1243 to −1239 region and TATTT in the −1232 to −1228 region, were responsible for the *R. solani*-inducible activity. These two *cis*-elements were also identified to have *R. solani*-specific-inducible activity in stable transgenic rice plants, suggesting the existence of a novel regulation mechanism involved in the interaction between *R. solani* and *Zea mays.*

Plants are frequently exposed to diverse biotic stresses that jeopardize their growth, development and metabolism. To ensure their survival, plants have evolved complicated and delicate sensing mechanisms and response pathways resulting in adaptive responses through physiological and morphological changes. The defense responses are triggered by invading pathogens or pathogen-derived elicitors, and eventually lead to extensive transcriptional reprogramming^1^. Numerous defense-related genes are up-regulated during plant immune responses and play important roles in disease resistance^2^,^3^,^4^. These findings suggest that the promoters of these genes may have *cis*-elements involved in the response to pathogens.

The identification of functional *cis*-acting elements in a promoter is a crucial step toward understanding gene function^5^. Therefore, much effort has been devoted to the characterization of *cis*-acting elements involved in pathogen- or fungal elicitor-induced defense gene expression, including the studies on the W-box^6^,^7^,^8^, GCC-box^9^,^10^, S-box^11^, MREs (MYB recognition elements)^12^, G-box^13^, E-box^14^, PRE2 and PRE4^15^. Some of these elements have been used to construct synthetic promoters, such as the W-box, GCC-box and S-box, and these synthetic promoters can contribute to pathogen inducibility^16^,^17^. Additionally, experiments have shown that synthetic promoters containing combinations of *cis*-acting elements are generally better than promoters containing just one type of element^18^. These promoters are valuable additions to the study of signaling and transcriptional activation during plant-pathogen interactions.

Banded leaf and sheath blight (BLSB), mainly caused by the fungal pathogen *Rhizoctonia solani*, is a highly devastating disease in most maize-growing areas of the world, and it is wide spread both in maize and rice^19^,^20^. The disease often leads to extensive necrosis in the leaf sheaths of hosts, eventually causing the death of the infected plant and resulting in substantial economic losses. Thus far, limited progress has demonstrated that sheath blight resistance is only controlled by minor effect QTLs^21^,^22^, and only a few genes, such as *Zmb-32*^23^, *OsRC7*^24^, *ZmMOD1* and *OsRCH10*^25^, *Oschi11*^26^, *Ostlp*^27^, *Os2H16*^28^, *Osoxo4*^29^, *OsACS2*^30^, *OsJERF1*^31^ and *OsWRKY4*^32^, were identified as resistant to *R. solani*. However, knowledge regarding the regulation mechanisms of *R. solani*-inducible genes is still very limited, with only a few *R. solani*-inducible promoters having been identified^33^,^34^,^35^, and no *R. solani*-inducible *cis*-elements yet reported.

In this study, we mined the *R. solani*-induced maize gene *GRMZM2G315431* from RNA sequencing (RNA-Seq) data. To better understand how the *GRMZM2G315431* gene is regulated, we characterized the *GRMZM2G315431* promoter region using the β-glucuronidase (*GUS*) gene as a reporter gene. Our results showed that the 5′-flanking sequence of the *GRMZM2G315431* promoter was induced by *R. solani*. Deletion analysis showed that two novel *cis*-elements, GTTGA and TATTT, were responsive to *R. solani* infection, with further analysis indicating that the two *cis*-elements were responsible for inducing the expression of reporter genes in response to *R. solani*, but not to other fungal and bacterial rice pathogens, *Magnaporthe grisea, Xanthomonas oryzae* pv. *oryzae (Xoo*) and *Xanthomonas oryzae* pv. *oryzicola (Xoc*). These results will increase our understanding of *GRMZM2G315431* regulation and increase the number of promoter and *cis*-elements available for potential use in both basic research and the development of transgenic plants with enhanced *R. solani* resistance.

## Results

### Identification of *R. solani*-inducible genes

**Figure 1:**
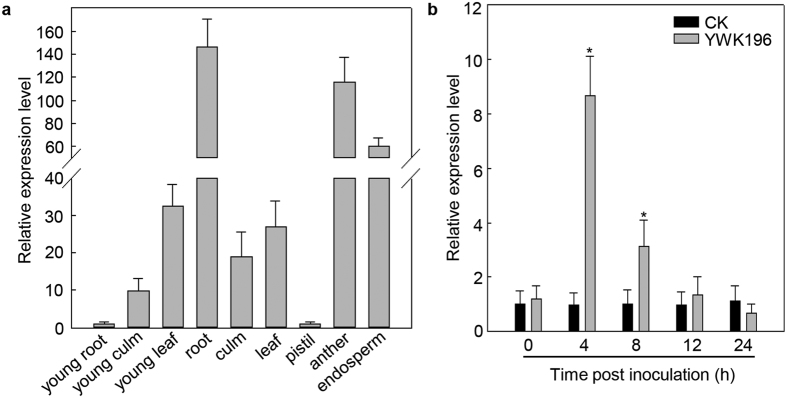
Expression analysis of the maize gene *GRMZM2G315431* by qRT-PCR. (**a**) Tissue-specific analysis of *GRMZM2G315431*. (**b**) Time course of *GRMZM2G315431* expression after *R. solani* infection in 21-day-old seedlings. Error bars indicate the SD (n = 3). Asterisks indicate P < 0.05 (*) in Student’s t test analysis.

To identify genes that are up-regulated by *R. solani*, RNA-Seq was used in the RNA expression profiling of *R. solani*-inoculated maize sheaths and uninoculated sheaths. Hundreds of genes were identified as induced by *R. solani* in the RNA-Seq data (Accession NO. SRP076058). *GRMZM2G315431* was one of the induced genes, and its expression patterns were further analyzed by qRT-PCR. As shown in Fig. 1a, *GRMZM2G315431* was expressed at higher levels in most tissues except the young root, young culm and pistil. An 8-fold increase in *GRMZM2G315431* expression was observed 4 h after inoculation with *R. solani* strain YWK196, with the expression levels then gradually decreasing to basal levels by 12 h (Fig. 1b), suggesting that the *GRMZM2G315431* promoter could respond to *R. solani*.

### Bioinformatic analysis of putative *cis*-elements in the *GRMZM2G315431* promoter

To characterize the regulatory mechanisms controlling transcription of the *GRMZM2G315431* gene, we isolated its promoter region (−1833 to +194), which extends into the 5′-untranslated region (Accession NO. KX507259). According to the PLACE database^36^, some putative *cis*-elements are predicted in pGRMZM2G315431 (see Supplementary Fig. S1). The region with the highest homology to a TATA-box consensus sequence (5′-TATAT-3′) starts 269 bp upstream of the ATG and 75 bp upstream of the transcription start point (TSP). In addition, no obvious CAAT box was found to be located close to the TATA-box on either strand. Four elements with homology to pathogen- and salt-inducible elements (GAAAAA) were located within the promoter^37^. The *GRMZM2G315431* promoter also contains one ethylene (ET) responsive element, AATTCAAA^38^, two gibberellic acid (GA) responsive elements, TAACAA^39^ and three abscisic acid (ABA) responsive elements, ACGTG^40^. On the opposite strand, we found one element that is complementary to a TGACG element. This element has been detected in constitutive root promoters in association with the *as-1* element^41^,^42^. Fifteen CANNTG elements, which are known to bind proteins belonging to the helix-loop-helix (bHLH) transcriptional regulator superfamily, were found in the promoter region^43^. There was also one W-box (TTGACT), which is specifically recognized by WRKY DNA binding proteins^44^, in the promoter.

### Analysis of tissue-specific and *R. solani*-inducible expression of the *GRMZM2G315431* promoter

**Figure 2:**
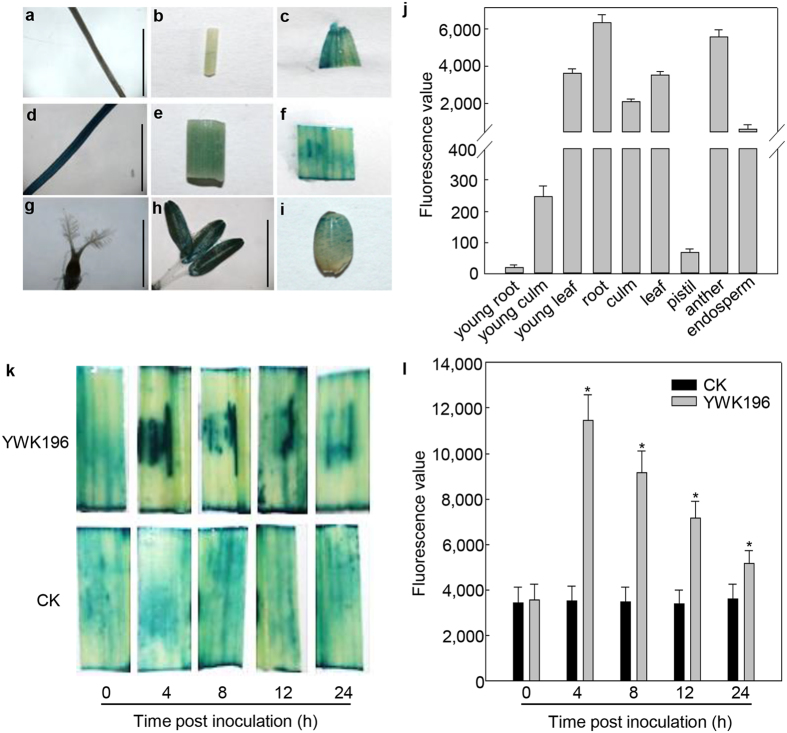
Expression pattern of the *GRMZM2G315431* promoter in transgenic rice plants. (**a**–**i**) GUS histochemical staining in different tissues of transgenic rice. (**a**) young root; (**b**) young culm; (**c**) young leaf; (**d**) root; (**e**) culm; (**f**) leaf; (**g**) pistil; (**h**) stamen; (**i**) endosperm. Bars = 5 mm. (**j**) Quantitative GUS assays of different tissues from transgenic rice. GUS activity was calculated relative to young root. (**k**) GUS histochemical staining in transgenic rice plants post inoculation with *R. solani* strain YWK196. (**l**) GUS activity in transgenic rice leaves treated as described in (**k**). GUS activity was calculated relative to 0 h of CK. Error bars indicate the SD (n = 3). Asterisks indicate P < 0.05 (*) in Student’s t test analysis.

First, we examined the tissue-specific expression pattern of a *GRMZM2G315431* promoter-*GUS* in transgenic rice to see whether it matched the expression pattern of the *GRMZM2G315431* gene in maize. As shown in Fig. 2a–j, the *GRMZM2G315431* promoter-*GUS* was expressed primarily in young leaf, root, culm, leaf, anther and endosperm. The GUS staining patterns in transgenic rice plants were similar to the expression patterns of *GRMZM2G315431* mRNA in maize (Fig. 1a).

We then examined the effect of *R. solani* on GUS reporter gene expression in transgenic rice leaves. After treatment with *R. solani* strain YWK196, the GUS activity of the *GRMZM2G315431* promoter-*GUS* was enhanced approximately 4-fold by 4 hours post inoculation (hpi), and slowly declined by 24 hpi, but remained higher than the control (Fig. 2k and l). The induction pattern was similar to the expression pattern found in maize (Fig. 1b). Overall, these experiments show a conserved *GRMZM2G315431* regulation mechanism in both maize and rice.

### Deletion analysis of the *GRMZM2G315431* promoter

**Figure 3:**
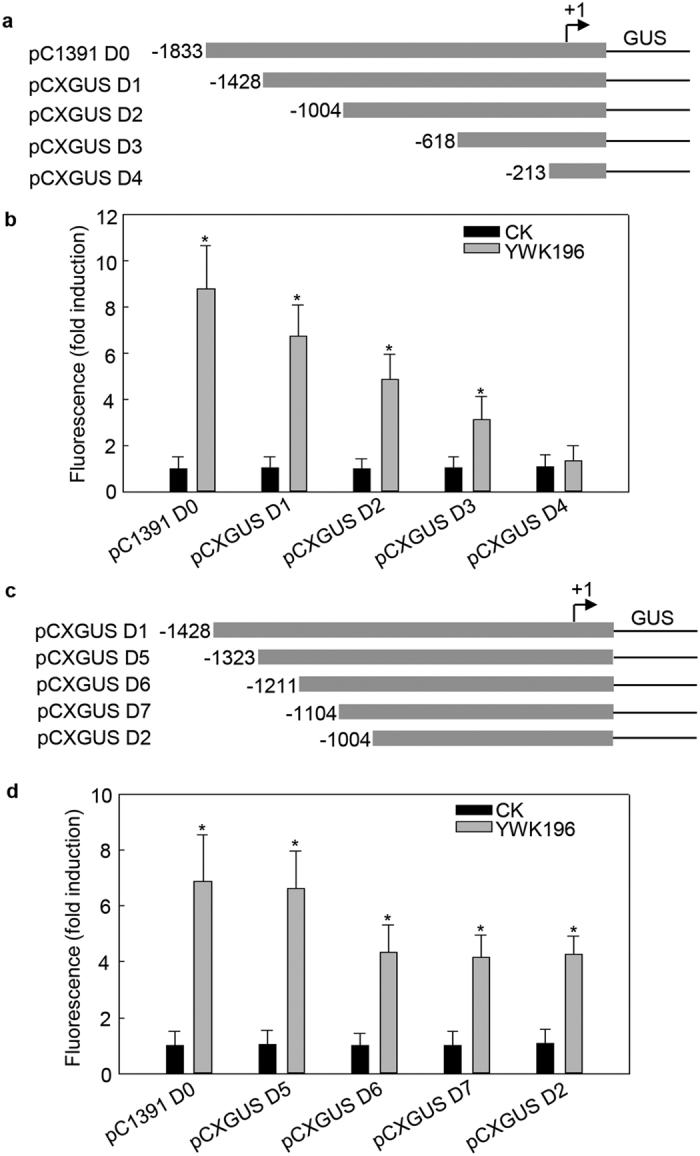
Quantitative fluorometric assays for GUS activity driven by various *GRMZM2G315431* promoter deletion constructs. (**a**) Diagram of various deletion derivatives of the *GRMZM2G315431* promoter. The deletion end points are indicated in bp from the transcription start site. All promoter derivatives were fused to a *GUS* reporter vector, *pCXGUS-P*. (**b**) The GUS activity of the DNA constructs prepared in (**a**) in a tobacco transient expression system. The tobacco leaves were inoculated with *R. solani* strain YWK196 for 24 h. GUS activity was calculated relative to CK of the pC1391 D0 construct. (**c**) Diagram of the deletion constructs in the −1428 to −1004 region of the *GRMZM2G315431* promoter. All promoter derivatives were fused to a *GUS* reporter vector, *pCXGUS-P*. (**d**) The GUS activity of DNA constructs prepared in **c** in a tobacco leaf transient expression system. The tobacco leaves were inoculated with *R. solani* strain YWK196 for 24 h. GUS activity was calculated relative to CK of the pCXGUS D1 construct. Error bars indicate the SD (n = 3). Asterisks indicate P < 0.05 (*) in Student’s t test analysis.

To determine the specific regions of the promoter that are involved in *GRMZM2G315431* induction by *R. solani* treatment, a series of 5′ deletions were made in the *GRMZM2G315431* promoter region (Fig. 3a). Each construct was transiently introduced into *Nicotiana benthamiana* leaves by *Agrobacterium tumefaciens*-mediated transformation, and GUS activity was assayed after treatment with YWK196 for 24 h. As shown in Fig. 3b, the highest level of inducible GUS activity was found in assays with the full-length pC1391 D0 construct. However, this induction weakened in constructs containing deletions up to −1428 (pCXGUS D1), −1004 (pCXGUS D2) and −618 (pCXGUS D3) in turn, but still remained higher than that in the control. In contrast, GUS inducible activity was completely lost in the construct containing a deletion up to −213 (pCXGUS D4). These results indicated that the deleted regions −1833 to −1429, −1428 to −1005, −1004 to −619 and −618 to −214 are involved in the response to *R. solani*, suggesting that putative *R. solani*-responsive elements exist in these regions. Bioinformatic analysis of the *GRMZM2G315431* promoter predicted that regions −1833 to −1429, −1004 to −619 and −618 to −214 contain the reported pathogen-inducible *cis*-element GT-1^37^, that the region −1004 to −619 contains one W-box and that the region −1428 to −1005 contains no pathogen-inducible *cis*-elements, suggesting the presence of uncharacteristic pathogen-inducible *cis*-elements in this region.

To mine the *cis*-elements within the −1428 to −1005 region that are responsible for induction by *R. solani* treatment, a series of 5′ deletions were made in this region (Fig. 3c). These constructs were then tested in transient expression assays in tobacco leaves treated for 24 h with *R. solani* strain YWK196 (Fig. 3d). The deletion up to −1323 construct (pCXGUS D5) showed GUS induction almost equal to that of the pCXGUS D4 construct. The construct containing the −1211 to +194 region (pCXGUS D6) showed a roughly one-third reduction in GUS activity compared with the pCXGUS D4 and pCXGUS D5 constructs, and the GUS induction level of the deletion up to −1104 (pCXGUS D7) and −1004 (pCXGUS D2) constructs was equal to that of the pCXGUS D6 construct. These results indicated that the −1323 to −1212 fragment is involved in the region −1428 to −1005 response to *R. solani*.

### The −1243 to −1228 fragment is crucial for the region −1323 to −1212 response to *R. solani*

**Figure 4:**
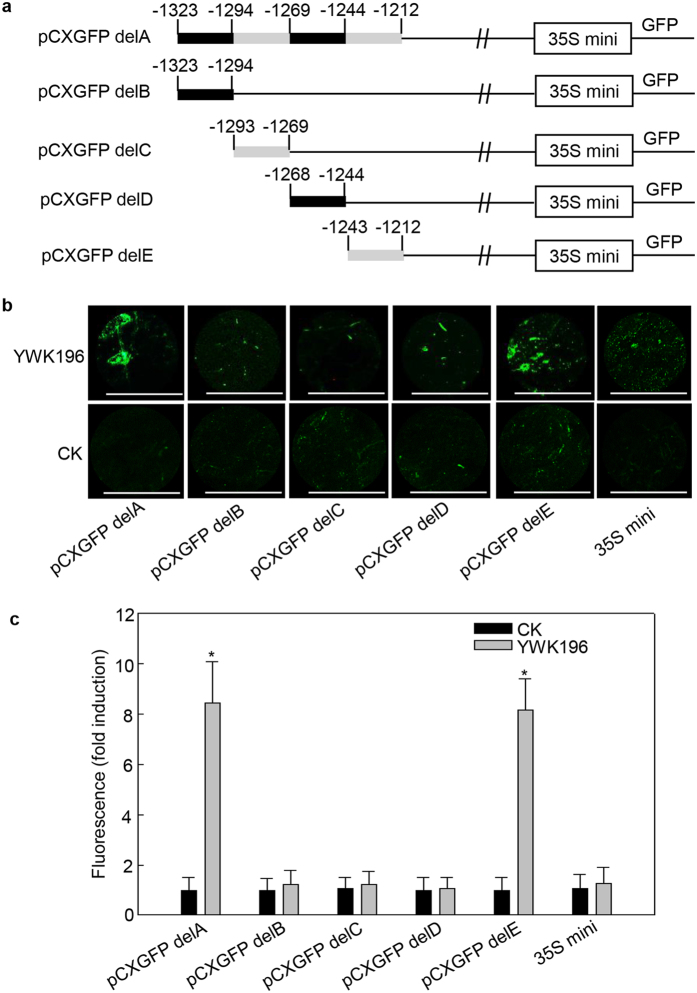
The −1243 to −1212 fragment of the −1323 to −1212 region is the core *R. solani*-responsive region. (**a**) Schematic diagram of the −1323 to −1212 region (pCXGFP delA) and the four deleted derivatives (pCXGFP delB to pCXGFP delE) used to express *GFP* in tobacco leaves. The white boxes represent the 35S minimum promoter. (**b**) GFP fluorescence assay of young and expanded symmetrical tobacco leaves infiltrated with pCXGFP delA or its derivatives after *R. solani* strain YWK196 infection for 24 h. GFP fluorescence was observed under a Leica M205 C stereo microscope. Bars = 5 mm. (**c**) Quantitative fluorometric assay of tobacco leaves prepared in (**b**). The fluorescence value was calculated relative to CK of pCXGFP delA. The 35S minimum promoter was used as the negative control. Error bars indicate the SD (n = 3). Asterisks indicate P < 0.05 (*) in Student’s t test analysis.

To identify the crucial region (s) within the −1323 to −1211 region that are responsible for induction by *R. solani* treatment, this region was further divided into four fragments of 20 to 30 bp in length, which were individually fused with the 35S minimum promoter-*GFP* contained within the *pCXGFP-P* vector (Fig. 4a). These constructs were then tested in transient expression assays in tobacco leaves treated for 24 h with *R. solani* strain YWK196 (Fig. 4b,c). The −1323 to −1212 construct (pCXGFP delA) and the −1243 to −1212 construct (pCXGFP delE) showed GFP induction of approximately 8-fold after treatment with YWK196. In contrast, the pCXGFP delB, pCXGFP delC and pCXGFP delD constructs exhibited a very faint fluorescence signal.

**Figure 5:**
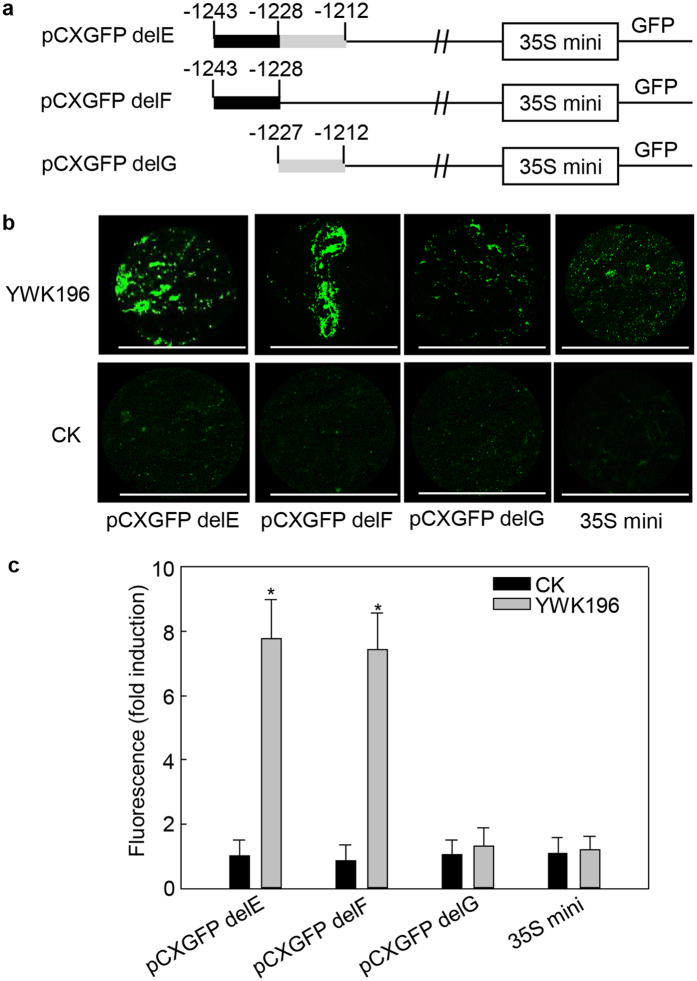
A 16 bp sequence is crucial for induction of the −1243 to −1212 fragment by *R. solani*. (**a**) Schematic diagram of the −1243 to −1212 region (pCXGFP delE) and the two deleted derivatives (pCXGFP delF and pCXGFP delG) used to express *GFP* in tobacco leaves. The white boxes represent the 35S minimum promoter. (**b**) GFP fluorescence assay of young and expanded symmetrical tobacco leaves infiltrated with pCXGFP delE or its derivatives after *R. solani* strain YWK196 infection for 24 h. GFP fluorescence was observed under a Leica M205 C stereo microscope. Bars = 5 mm. (**c**) Quantitative fluorometric assay of tobacco leaves prepared in (**b**). The fluorescence value was calculated relative to CK of pCXGFP delE. The 35S minimum promoter was used as the negative control. Error bars indicate the SD (n = 3). Asterisks indicate P < 0.05 (*) in Student’s t test analysis.

To narrow the region that is responsive to *R. solani*, we generated two 5′-deletion derivatives of the −1243 to −1212 region that were individually fused with the 35S minimum promoter-*GFP* contained within the *pCXGFP-P* vector (Fig. 5a). The resulting constructs pCXGFP delF and pCXGFP delG were used for the tobacco transient expression assay. As shown in Fig. 5b and c, the pCXGFP delF construct showed an almost equal level of GFP induction (approximately 8-fold) compared to that of the pCXGFP delE construct after inoculation with YWK196. In contrast, no considerable GFP signal was detected in the leaves expressing the pCXGFP delG construct. Overall, these experiments suggest that a 16 bp sequence (GTTGAGGCACTTATTT) containing potential novel *cis*-acting elements is responsive to *R. solani*.

### Two novel *cis*-elements are the core elements involved in the −1243 to −1228 region response to *R. solani*

**Figure 6:**
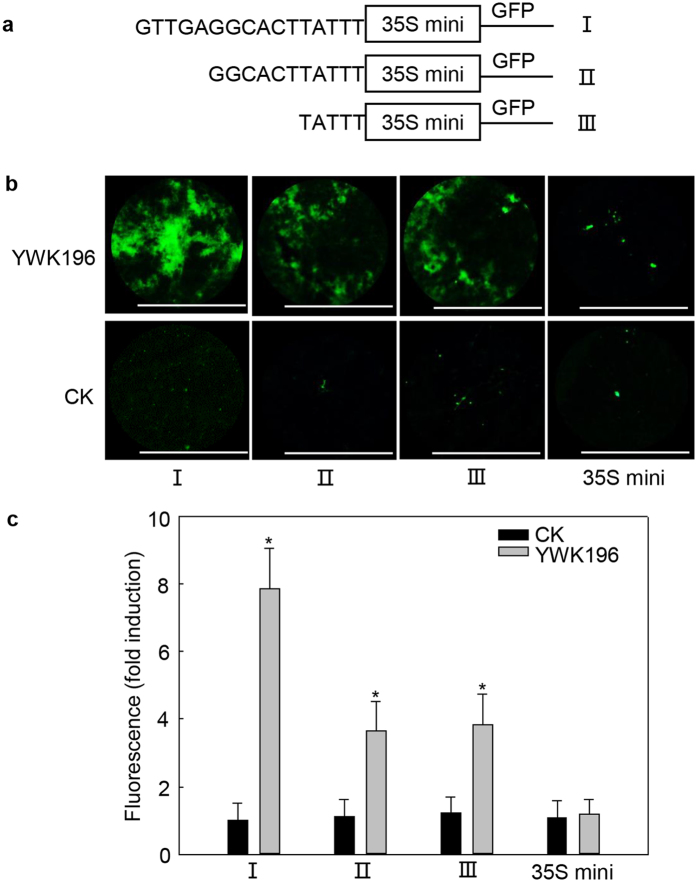
The GTTGA (−1243 to −1239) and TATTT (−1232 to −1228) sequences are two novel *cis*-elements confer induction by *R. solani*. (**a**) Schematic diagram of the 16 bp sequence (I) and the two deleted derivatives (II and III) used to express *GFP* in tobacco leaves. The white boxes represent the 35S minimum promoter. (**b**) GFP fluorescence assay of young and expanded symmetrical tobacco leaves infiltrated with the three constructs prepared in (**a**) after *R. solani* strain YWK196 infection for 24 h. GFP fluorescence was observed under a Leica M205 C stereo microscope. Bars = 5 mm. (**c**) Quantitative fluorometric assay of tobacco leaves prepared in (**b**). Fluorescence value was calculated relative to CK of I. The 35S minimum promoter was used as the negative control. Error bars indicate the SD (n = 3). Asterisks indicate P < 0.05 (*) in Student’s t test analysis.

To identify the core element involved in the response to *R. solani* stress, we further produced a series of deletion derivatives fused with the 35S minimum promoter-*GFP* focusing on the identified 16 bp sequence (Fig. 6a). Transient expression assays showed that deletion of the GGTGA sequence reduced the induction level by more than one-half compared with the induction of the full 16 bp sequence. However, deletion of the sequence GGTGAGGCACT exhibited an induction level (3.6-fold) nearly equal to that of the GGTGA deletion, with the remaining TATTT sequence maintaining this level (Fig. 6b,c). These results indicated that GGTGA and TATTT are the two *cis*-elements that play roles in the *R. solani* induction of the 16 bp sequence.

**Figure 7:**
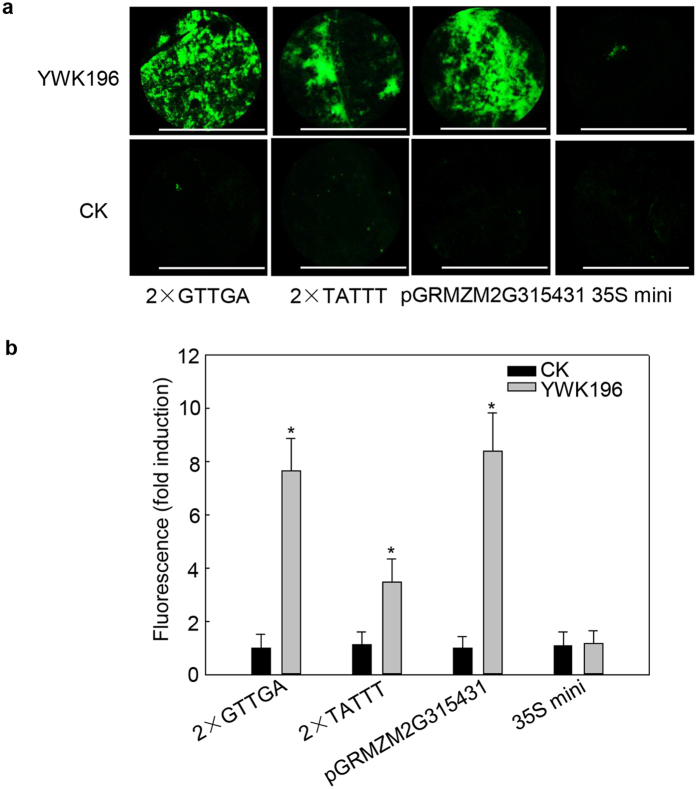
Determination of GTTGA and TATTT induction by *R. solani*. (**a**) GFP fluorescence assay of tobacco leaves expressing 2 × GTTGA and 2 × TATTT after treatment with *R. solani* strain YWK196 for 24 h. GFP fluorescence was observed under a Leica M205 C stereo microscope. Bars = 5 mm. (**b**) Quantitative fluorometric assay of tobacco leaves prepared in (**a**). The fluorescence value was calculated relative to CK of 2 × GTTGA. The full-length *GRMZM2G315431* promoter and 35S minimum promoter were used as the positive and negative controls, respectively. Error bars indicate the SD (n = 3). Asterisks indicate P < 0.05 (*) in Student’s t test analysis.

To further determine whether these two *cis*-element could respond to *R. solani* independently, we produced two constructs in which two tandem repeats of the two *cis*-elements (2 × GGTGA and 2 × TATTT) were fused with the 35S minimum promoter-*GFP*. The full-length *GRMZM2G315431* promoter and empty vector were used as controls. As shown in Fig. 7, relatively high (7.5-fold) induction of 2 × GGTGA suggested that two tandem repeats of GGTGA alone are almost sufficient for full *R. solani* induction of the *GRMZM2G315431* promoter (8.6-fold). However, *R. solani* induced 2 × TATTT to a level approximately one-half that of the *GRMZM2G315431* promoter and showed no substantial difference from the induction of a single TATTT, suggesting that multiplicating TATTT elements could not promote its response to *R. solani*. Taken together, these data indicated that GGTGA and TATTT are two *R. solani*-inducible *cis*-elements.

### Transgenic rice analysis confirmed the results of the tobacco transient-expression assay

**Figure 8:**
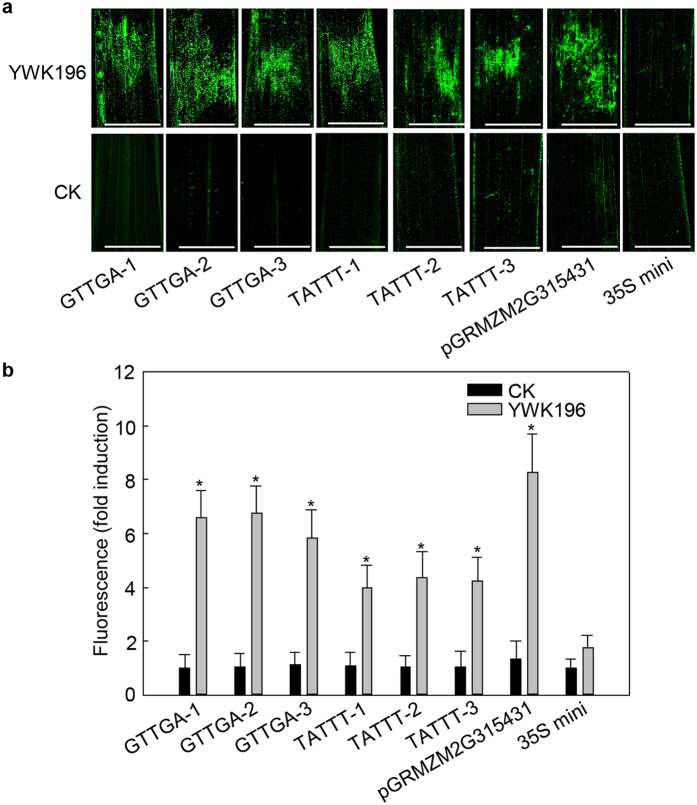
GFP expression driven by GTTGA and TATTT in the transgenic rice leaves. (**a**) GFP fluorescence assay of transgenic rice leaves inoculated with *R. solani* strain YWK196. Three T_1_ lines of each element were used. Bars = 5 mm. (**b**) Quantitative fluorometric assay of transgenic rice leaves post inoculation with *R. solani*. The fluorescence value was calculated relative to CK of GTTGA-1. The full-length *GRMZM2G315431* promoter and 35S minimum promoter were used as the positive and negative controls, respectively. Error bars indicate the SD (n = 3). Asterisks indicate P < 0.05 (*) in Student’s t test analysis.

While transient assays might provide some insight into the regulation of a promoter element outside its native context, transient-driven expression patterns do not always correlate with the expression patterns observed in stable events. To further confirm the results of aforementioned transient assays, we generated GGTGA and TATTT transgenic rice plants using the constructs produced in Fig. 7. Three T_1_ lines of each element were inoculated with *R. solani* strain YWK196 for 24 h. Leaves covered with PDA medium were used as controls. As shown in Fig. 8, strong activation of the *GFP* gene was detected in the *R. solani*-inoculated leaves of GTTGA and TATTT transgenic rice and weak expression was observed in the mock leaves. Additionally, the two tandem repeats of GGTGA were nearly sufficient for full *R. solani* induction of the *GRMZM2G315431* promoter, while the induction level of 2 × TATTT was approximately one-half of the induction of the *GRMZM2G315431* promoter. These results were consistent with those of the tobacco transient-expression assays.

To test whether the two *cis*-elements were responsive to other *R. solani* strains and other rice pathogens, both transgenic lines used above were inoculated with *R. solani* strains LD16, LD21, *M. grisea, Xoo* strain PXO99 and *Xoc* strain RS105 for 24 h. Leaves treated with medium and water were used as controls, respectively. As shown in Supplementary Fig. S2, GFP activation was detected only in LD16- and LD21-inoculated leaves, while no considerable GFP was detected in leaves infected by other pathogens. These results indicate that the two *cis*-elements are not responsible for inducing the expression of reporter genes in response to *M. grisea, Xoo* or *Xoc.* Therefore, these *cis*-elements might be *R. solani*-specific-inducible elements.

## Discussion

The transcriptional regulation of genes is orchestrated by their corresponding promoters in response to myriad biotic and abiotic factors. Each gene utilizes specific *cis*-acting elements in its 5′ regulatory region that control different biological processes and/or stress responses. Therefore, it is very important to identify and characterize functional *cis*-elements to understand transcriptional regulation and subsequently facilitate plant genetic engineering.

Currently, most of the genes used in transgenic crops are driven by constitutive promoters^45^. Although the production of transgenic plants with enhanced stress tolerance has succeeded, the constitutive expression of transgenes sometimes leads to undesirable effects on growth, development and crop yield^45^. A solution to this problem is the use of stress-inducible promoters, which allow the controlled expression of target genes at certain stages of plant development or during stress episodes. The use of inducible promoters may restrict the product of a transgene to the cells surrounding the pathogen-infection site and prevent expression in healthy parts of the plant^46^,^47^. Thus, studies on pathogen-inducible promoters, particularly their *cis*-acting elements, could have potential application to the engineering of crops with increased disease resistance.

In this study, we mined the *R. solani*-induced maize gene *GRMZM2G315431* from previous RNA-Seq data. We prepared transgenic rice carrying *GRMZM2G315431* promoter-*GUS* construct to understand the upstream signaling mechanisms responsible for *GRMZM2G315431* gene expression in response to *R. solani*, and found that GUS was expressed in young culm, young leaf, root, culm, leaf, anther and endosperm but not in young root and pistil (Fig. 2a–j). This finding is consistent with the results of a qRT-PCR analysis of maize (Fig. 1a). This result strongly suggests that the expression patterns of the *GRMZM2G315431* promoter are maintained in heterologous transgenic rice plants.

To further determine the key *cis*-element required for the *R. solani*-inducible activity of pGRMZM2G315431, we tested a series of deleted pGRMZM2G315431 derivatives and identified several *cis*-acting elements that confer *R. solani* inductivity. GT-1 (GAAAAA) has been reported as a pathogen-inducible *cis*-acting element that can be induced by *Pseudomonas syringae*^37^. Our results showed that a total of four GT-1 elements are located in the −1833 to −1429, −1004 to −619 and −618 to −214 regions (see Supplementary Fig. S1). Deletion of three regions reduced pGRMZM2G315431 induction by *R. solani* (Fig. 3b), suggesting that GT-1 may be involved in the induction of these regions by *R. solani*. These results also revealed that GT-1 is involved in a conserved signaling pathway that responds to infection by different pathogens. Additionally, the −1004 to −619 region contains one W-box (TTGACT) element. The W-box has been reported to specifically bind to WRKY proteins and is involved in defense-related WRKY regulation^44^,^48^,^49^,^50^. In the −1248 to −1005 region, we identified two *cis*-elements, GTTGA and TATTT, which are necessary for the *R. solani* induction (Fig. 6). The GTTGA sequence has been reported as a MexR binding site from *P. aeruginosa*^51^. The other sequence, TATTT, is very similar to the core sequence of the TATA box. The TATA box is usually located 25–35 bp upstream of the transcription start site^52^, while the TATTT sequence in our study was located 1034 bp upstream of the transcription start site. Additionally, the TATTT sequence has been identified as an important motif in the *ica* locus promoter of *Staphylococcus aureus*, which has a role in biofilm production^53^. However, there are no reports regarding the functional roles of these two sequences in plants. Here, we showed that the GTTGA and TATTT sequences are responsible for inducing expression in response to the fungal pathogen *R. solani* but not to other rice pathogens *M. grisea, Xoo* and *Xoc* (see Supplementary Fig. S2), suggesting that these two pathogen-inducible elements may be *R. solani*-specific and could be further used to improve crop resistance to *R. solani* specifically. These results also reveal that the two *cis*-elements are different from the pathogen-inducible *cis*-elements that were involved in basal immunity. Further investigation into the regulation of *GRMZM2G315431* will involve the characterization of interacting transcription factors, which will provide a better understanding of the roles played by DNA-protein interactions in *GRZM2G315431* gene expression during the maize-*R. solani* interaction and supply novel resources in the form of synthetic promoters and transcription factors for the genetic improvement of *R. solani* resistance in crops.

## Methods

### Plant materials and treatments

Maize (*Zea mays* L., B73) and rice (*Oryza sativa* L. cv. japonica, Zhonghua 11) plants were grown at 28 °C with a 16/8 h light/dark cycle. Tobacco (*Nicotiana benthamiana*) was grown at 25 °C under a 16/8 h light/dark cycle. For the tissue expression analysis of *GRMZM2G315431* and its promoter, young root, young culm, young leaf, root, culm, leaf, anther, pistil and endosperm were harvested for total RNA isolation and the GUS assay. For the pathogen-inducible analysis of *GRMZM2G315431* and its promoter, the *R. solani* strains were grown in Potato-Dextrose-Broth medium (polypeptone at 10 g/L, glutamic acid at 1 g/L, sucrose at 10 g/L and agar at 15 g/L) at 25 °C for 3 days. *M. grisea* was grown in Rice Bran medium (rice bran at 20 g/L, yeast powder at 2 g/L and agar at 15 g/L) at 25 °C for 10 days. Inoculation of *R. solani* was performed using toothpicks as described previously^54^. Infected and non-infected plants were then harvested at 4, 8, 12 and 24 hpi for total RNA isolation and the GUS assay. *Xoo* strain PXO99 and *Xoc* strain RS105 were grown in Polypeptone-Sucrose-Agar medium (polypeptone at 10 g/L, glutamic acid at 1 g/L, sucrose at 10 g/L and agar at 15 g/L) at 28 °C for 2 days and then suspended in sterile water to OD_600_ = 0.5. Plants were inoculated with the PXO99 and RS105 suspensions by syringe infiltration at the seedling stage. Leaves were harvested for the GFP assay.

### Vector construction of the *GRMZM2G315431* promoter and its deletion derivatives

The full length *GRMZM2G315431* promoter containing the −1833 to +194 fragment was amplified from the maize inbred line B73 using the primers listed in Supplementary Table S1. To generate the promoter expression construct, the appropriate restriction sites were introduced into the PCR-amplified promoter (*Pst*I at the 5′ end; *Sal*I at the 3′ end). The PCR-amplified promoter was then cloned into *Pst*I/*Sal*I-digested *pCAMBIA1391* to obtain the expression construct, which was named pC1391 D0. The deleted promoters were cloned into the *pCXGUS-P* and *pCXGFP-P* vectors^55^ to generate deletion constructs containing various fragments (−1428 to +194, pCXGUS D1; −1004 to +194, pCXGUS D2; −618 to +194, pCXGUS D3; −213 to +194, pCXGUS D4; −1323 to +194, pCXGUS D5; −1211 to +194, pCXGUS D6; −1104 to +194, pCXGUS D7; −1323 to −1212, pCXGFP delA; −1323 to −1294, pCXGFP delB; −1293 to −1269, pCXGFP delC; −1268 to −1244, pCXGFP delD; −1243 to −1212, pCXGFP delE; −1243 to −1228, pCXGFP delF; −1227 to −1212, pCXGFP delG). To characterize the GTTGA and TATTT *cis*-elements, the two elements were repeated twice, ligated into the region upstream of the 35S minimum promoter within the *pCXGFP-P* vector and named 2 × GTTGA and 2 × TATTT, respectively.

### Rice transformation

For rice transformation, embryonic callus derived from mature embryos was infected with *Agrobacterium tumefaciens* strain EHA105^56^. For promoter analysis, pC1391 D0, 2 × GTTGA and 2 × TATTT constructs were transformed into rice cultivar Zhonghua 11 by *A. tumefaciens*-mediated transformation.

### Transient expression in tobacco and quantification of GUS and GFP

Promoter analysis was performed by transient expression in tobacco leaves according to a previously described method^57^. Histochemical GUS staining of transgenic rice leaves was performed as described^58^. The leaves were immersed in 0.1 M sodium phosphate buffer (pH = 7.0) containing 1 mg/ml X-Gluc, 0.5 mM K_3_[Fe(CN)_6_], 0.5 mM K_4_[Fe(CN)_6_], 10 mM Na_2_EDTA, 0.1% (v/v) Triton X-100, and 10% (v/v) methanol for 24 h at 37 °C in the dark. Quantitative fluorometric GUS assays were performed by incubating the extracts with the 4-methyl-umbelliferyl-β-D-glucuronide (MUG) substrate in a lysis buffer for 15 min at 37 °C. GFP fluorescence was observed under a Leica M205C stereo microscope, and fluorescence was quantified using a Synergy 2 Multi-Mode Reader as described in a previous method^59^. GFP fluorescence was excited at 480 nm and measured at 520 nm.

### RNA isolation and qRT-PCR analysis

Total RNA was isolated from 100 mg maize tissues using TRIzol reagent according to the manufacturer’s instructions (Invitrogen, Carlsbad, CA, USA). For cDNA synthesis, we used 2 μg of total RNA as a template and the SuperQuickRT MasterMix Kit (CWBIO, Beijing, China) in a 20 μL reaction mixture. Quantitative real-time PCR was performed with an UltraSYBR Mixture Kit (CWBIO, Beijing, China). *Actin1* (Accession NO.: GQ339773) mRNA levels were used to normalize the expression of each gene. Changes in expression were calculated using the ΔΔ Ct method. The gene-specific primers used are listed in Supplementary Table S1.

### Statistical analysis

All data analyses were repeated at least three independent times with three replicate experiments. Standard deviations were checked visually by error bars and the statistical significances were determined using an analysis of variance method. The data were subjected to one-way variance analysis, the mean differences were compared using Student’s t test, and P values < 0.05 were considered significant.

## Additional Information

**How to cite this article**: Li, N. *et al*. Identification of two novel *Rhizoctonia solani*-inducible *cis*-acting elements in the promoter of the maize gene, *GRMZM2G315431. Sci. Rep.* **7**, 42059; doi: 10.1038/srep42059 (2017).

**Publisher's note:** Springer Nature remains neutral with regard to jurisdictional claims in published maps and institutional affiliations.

## Supplementary Material

Supplementary Information
