# Supplementary material for: Identification of two novel Rhizoctonia solani-inducible cis-acting elements in the promoter of the maize gene, GRMZM2G315431
Source: Sci Rep. 2017 Feb 6;7:42059. doi: 10.1038/srep42059 (PMC5292686; doi:10.1038/srep42059)
Supplement: Supplementary Information [file srep42059-s1.pdf]

Identification of two novel *Rhizoctonia solani*-inducible *cis*-acting elements in the promoter of the maize gene, *GRMZM2G315431*

Ning Li[1,3], Jing Chen[2,3], Fangfang Yang[1], Shutong Wei[2], Lingguang Kong[2], Xinhua Ding[2]*, Zhaohui Chu[1]*

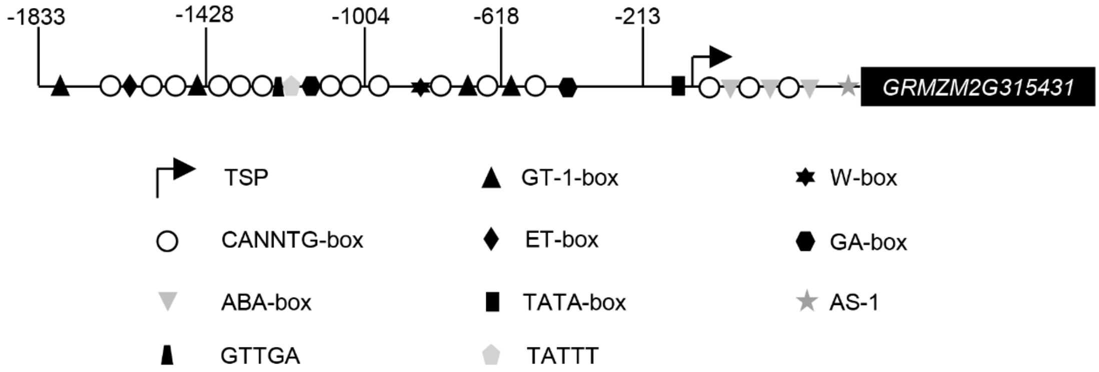

Supplementary Figure S1. Schematic map of the *GRMZM2G315431* promoter with putative *cis*-elements and two novel identified *R. solani*-inducible *cis*-elements. TSP, transcription start point; CANNTG-box, nematode-responsive box; ABA-box, ABA-responsive element; GT-1-box, pathogen- and NaCl-responsive element; ET-box, ET-responsive element; W-box, elicitor-responsive element; GA-box, GA-responsive element; AS-1, box of the *CaMV 35S* promoter; GTTGA and TATTT, two novel identified *R. solani*-inducible *cis*-elements.

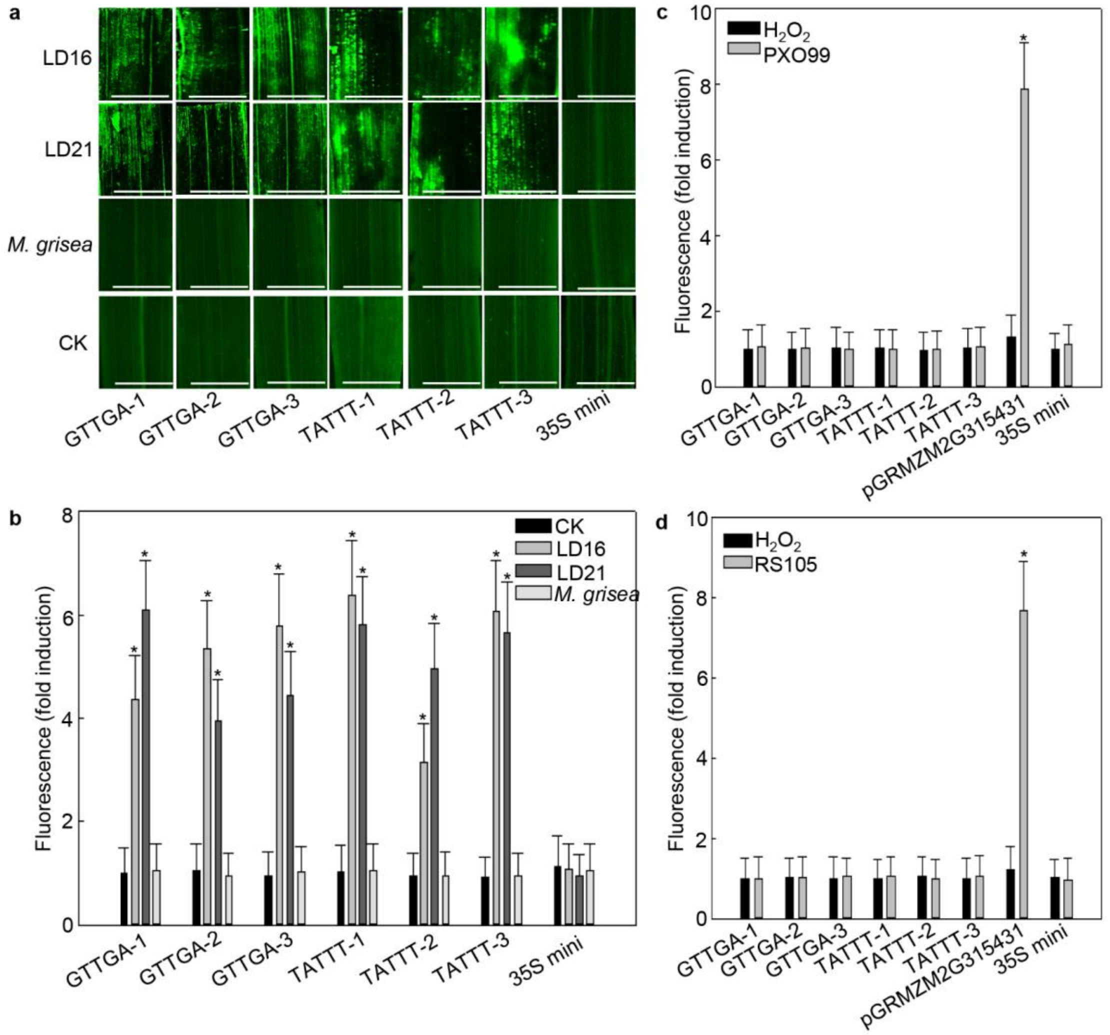

Supplementary Figure S2. GFP expression driven by GTTGA and TATTT in the transgenic rice leaves post inoculation with *R. solani* strains LD16, LD21, *M. grisea*, *Xoo* and *Xoc*. (**a**) GFP fluorescence assay of transgenic rice leaves inoculated with *R. solani* strains LD16, LD21 and *M. grisea*. Three T_1 lines of each element were used. Bars = 5 mm. (**b**) Quantitative fluorometric assay of transgenic rice leaves post inoculation with *R. solani* strains LD16, LD21 and *M. grisea*. The fluorescence value was calculated relative to CK of GTTGA-1. The 35S minimum promoter were used as the negative control. **(c)** Three T_1 lines of each element were inoculated with *Xoo* strain PXO99. The fluorescence value was calculated relative to H_2O_2 of GTTGA-1. The full-length *GRMZM2G315431* promoter and 35S minimum promoter were used as the positive and negative controls, respectively. (**d**) Three T_1 lines

of each element were inoculated with *Xoc* strain RS105. The fluorescence value was calculated relative to H_2O_2 of GTTGA-1. The full-length *GRMZM2G315431* promoter and 35S minimum promoter were used as the positive and negative controls, respectively. Error bars indicate the SD (n=3). Asterisks indicate $P < 0.05$ (*) in Student's t test analysis.

Table S1. List of primers used for expression analysis and cloning purposes. Restriction site sequences are underlined.

Primer name	Forward primer(5´-3´)	Reverse primer(5´-3´)
GRMZM2G315431	CGCGGTGCTCATCAACAG	CGTCGGTCTGGTCGAACAG
pC1391 D0	AAGCTGCAGTTCTATGGCAAAATCAATGAAGG	AAGGTCGACTGGCGGTGACGATGGTAA
pCXGUS D1	GAAAGATAGTGACCACTTTGACA	TGGCGGTGACGATGGTA
pCXGUS D2	CTATTTAGCTATACTTCATGCTGTT	TGGCGGTGACGATGGTA
pCXGUS D3	TGATTCACCTGCTGCTTATTTT	TGGCGGTGACGATGGTA
pCXGUS D4	GGTGTGAATCCGTATTCGAGACT	TGGCGGTGACGATGGTA
pCXGUS D5	AACAGTAGCATCTGAATCTGCAT	TGGCGGTGACGATGGTA
pCXGUS D6	AGGGCTTCAAACAAAGGTATT	TGGCGGTGACGATGGTA
pCXGUS D7	ATTTATTTCAGACCCAAAAGATT	TGGCGGTGACGATGGTA
pCXGFP delA	AACAGTAGCATCTGAATCTGCAT	TTATATAGAGGAAGGGTCTTGCGAATCCAATCTTTGGTGCAAATA
pCXGFP delB	AACAGTAGCATCTGAATCTGCATTCAGTGCGCAAGACCCTTCCTCTATATAA	GGGACTGACCTACCCGGG
pCXGFP delC	CTGGAGGAAGAGTTGTTAACAAGTACGCAAGACCCTTCCTCTATATAA	GGGACTGACCTACCCGGG
pCXGFP delD	TCGTAGCCGTTTGGACCCTCAGGTGCGCAAGACCCTTCCTCTATATAA	GGGACTGACCTACCCGGG
pCXGFP delE	GTTGAGGCACTTATTTGCACCAAAGATTGGATTCGCAAGACCCTTCCTCTATATAA	GGGACTGACCTACCCGGG
pCXGFP delF	GTTGAGGCACTTATTTCGCAAGACCCTTCCTCTATATAA	GGGACTGACCTACCCGGG
pCXGFP delG	GCACCAAAGATTGGATTCGCAAGACCCTTCCTCTATATAA	GGGACTGACCTACCCGGG
I	GTTGAGGCACTTATTTCGCAAGACCCTTCCTCTATATAA	GGGACTGACCTACCCGGG
II	GGCACTTATTTCGCAAGACCCTTCCTCTATATAA	GGGACTGACCTACCCGGG
III	TATTTCGCAAGACCCTTCCTCTATATAA	GGGACTGACCTACCCGGG
2×GTTGA	GTTGAGTTGACGCAAGACCCTTCCTCTATATAA	GGGACTGACCTACCCGGG
2×TATTT	TATTTTATTTCGCAAGACCCTTCCTCTATATAA	GGGACTGACCTACCCGGG
35S mini	CGCAAGACCCTTCCTCTATATAA	GGGACTGACCTACCCGGG
